# Supplementary material for: Decoration of the enterococcal polysaccharide antigen EPA is essential for virulence, cell surface charge and interaction with effectors of the innate immune system
Source: PLoS Pathog. 2019 May 2;15(5):e1007730. doi: 10.1371/journal.ppat.1007730 (PMC6497286; doi:10.1371/journal.ppat.1007730)
Supplement: S1 Fig — A cell suspension in phosphate saline buffer was adjusted to an OD at 600 nm of 1 and 1.5 μl of serial dilutions were spotted on BHI-agar plates containing various concentrations of lysozyme. ND, undiluted cell suspension; 10−1, 10-fold dilution; 10−2, 100-fold dilution; 10−3, 1000-fold dilution; 10−4, 10000-fold dilution; 10−5, 100000-fold dilution. (PPTX) [file ppat.1007730.s001.pptx]

## Slide 1
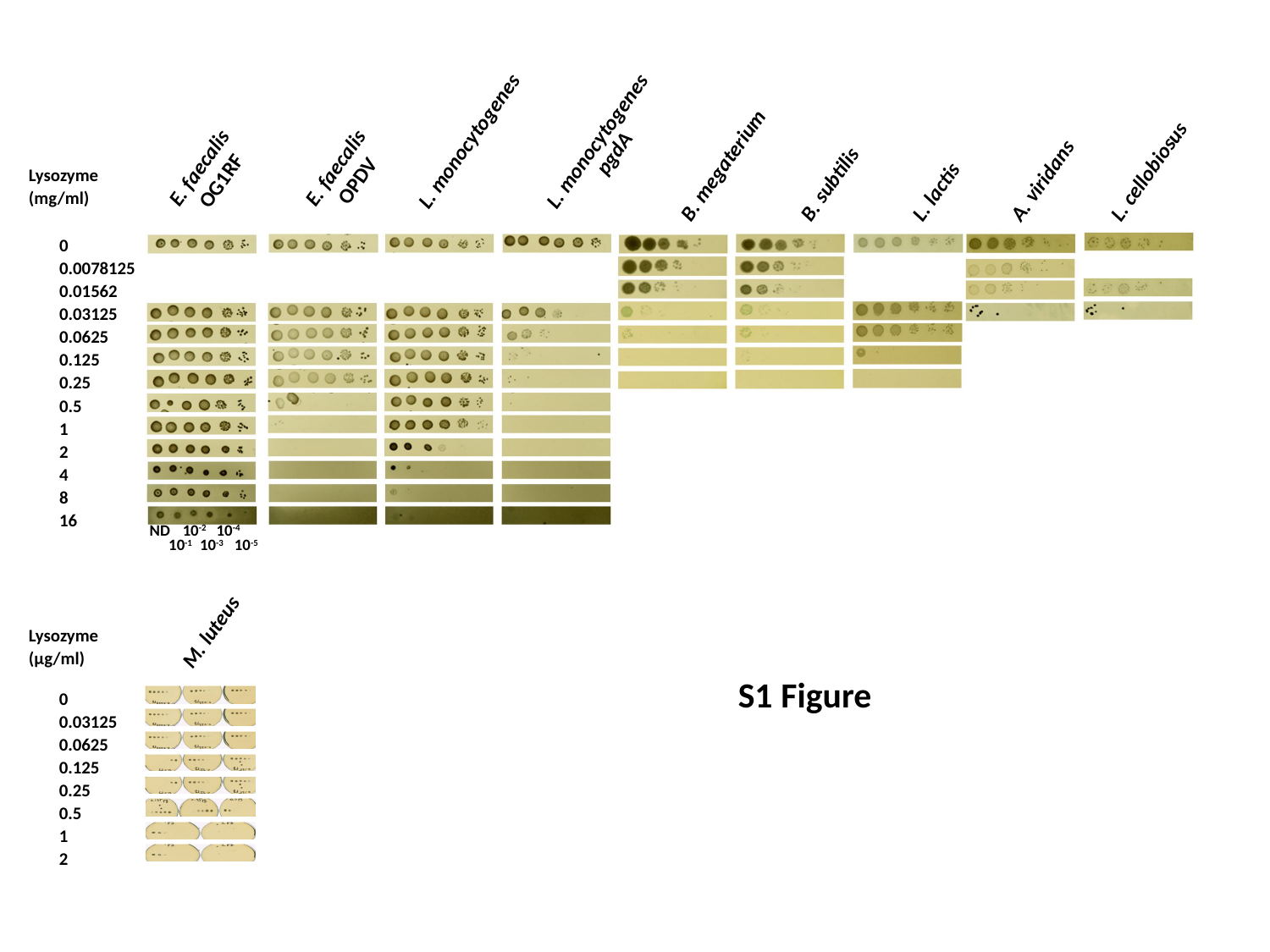

L. monocytogenes
L. monocytogenes
pgdA
E. faecalis
OG1RF
E. faecalis
OPDV
B. megaterium
L. cellobiosus
Lysozyme
(mg/ml)
A. viridans
B. subtilis
L. lactis
0
0.0078125
0.01562
0.03125
0.0625
0.125
0.25
0.5
1
2
4
8
16
ND 10-2 110-4
10-1 10-3-1 10-5
M. luteus
Lysozyme
(µg/ml)
S1 Figure
0
0.03125
0.0625
0.125
0.25
0.5
1
2
