# Supplementary figures and images for: Decoration of the enterococcal polysaccharide antigen EPA is essential for virulence, cell surface charge and interaction with effectors of the innate immune system
Source: PLoS Pathog. 2019 May 2;15(5):e1007730. doi: 10.1371/journal.ppat.1007730 (PMC6497286; doi:10.1371/journal.ppat.1007730)

## Slide 1
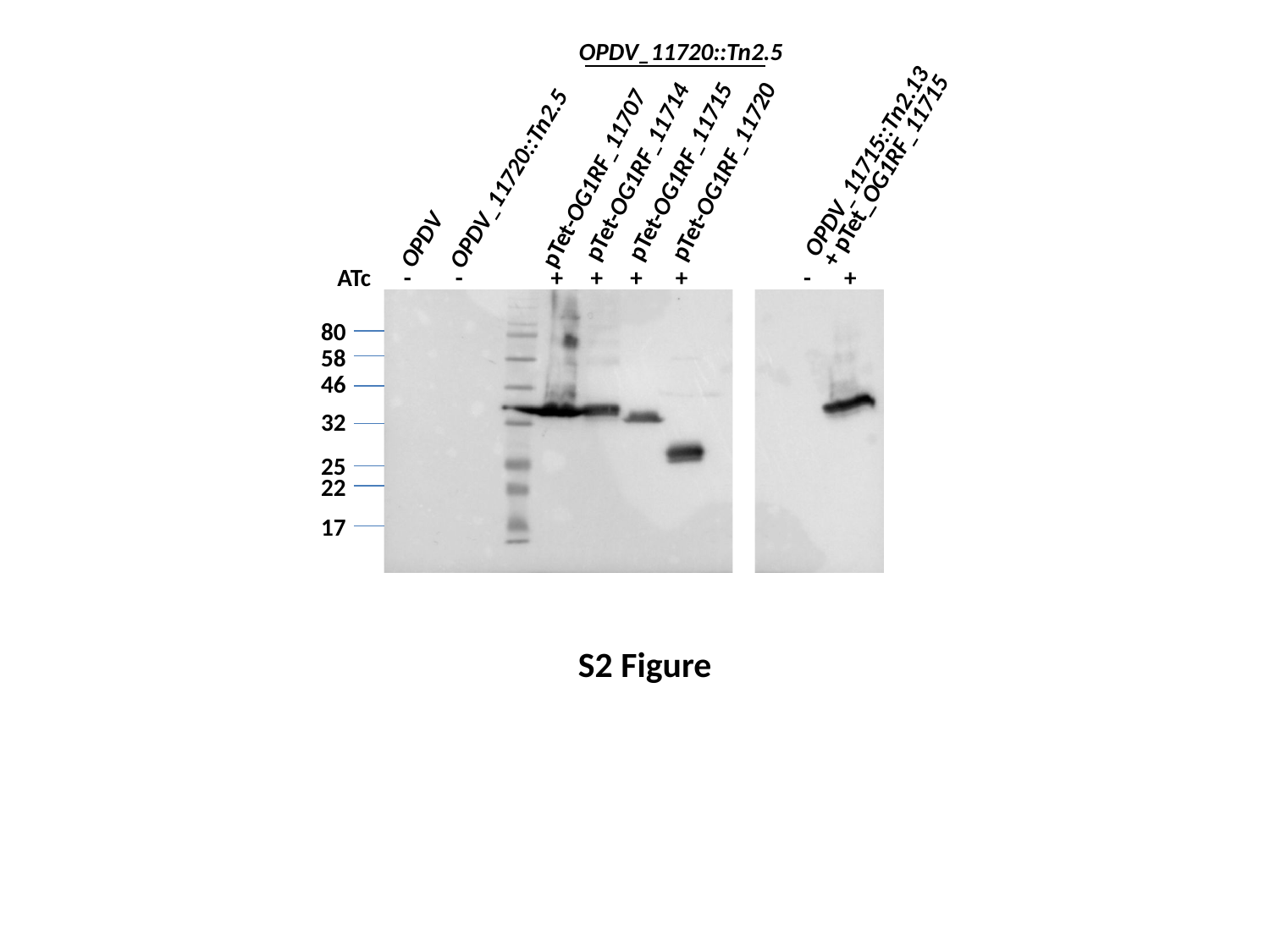

OPDV_11720::Tn2.5
OPDV_11715::Tn2.13
+ pTet_OG1RF_11715
pTet-OG1RF_11714
pTet-OG1RF_11715
pTet-OG1RF_11720
pTet-OG1RF_11707
OPDV_11720::Tn2.5
OPDV
ATc - - + + + +
 - +
80
58
46
32
25
22
17
S2 Figure

Supplement: S2 Fig — Cultures were grown in BHI to an OD at 600 nm of 0.5 and expression of the epa genes was induced by addition of anhydrotetracycline (10 ng ml-1). After 2 h, cells were harvested and mechanically broken in the presence of glass beads. Crude extracts (20 μg) were loaded on SDS-PAGE, transferred onto a nitrocellulose membrane and probed with a polyclonal serum against the polyhistidine tag. Bands of the expected molecular weights were detected (OG1RF_11707, 36.7 kDa; OG1RF_11714, 38.9 kDa; OG1RF_11715, 38.4 kDa; OG1RF_11720, 30.8 kDa). (PPTX) [file ppat.1007730.s002.pptx]

**A**

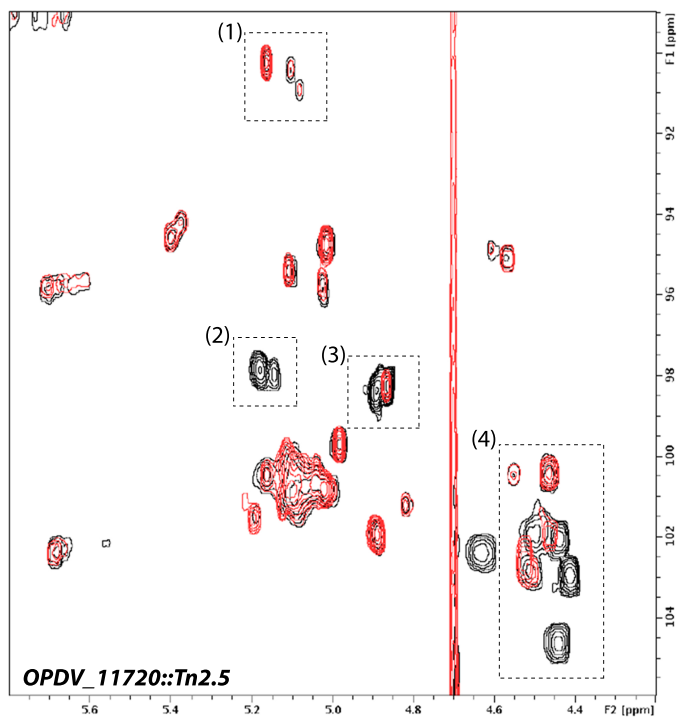

**B**

OPDV\_11720::Tn2.5    OPDV\_11720::Tn2.5    OPDV\_11720::Tn2.13    OPDV\_11720::Tn2.14    OPDV\_11720::Tn2.8  
+pTet-OG1RF\_11720

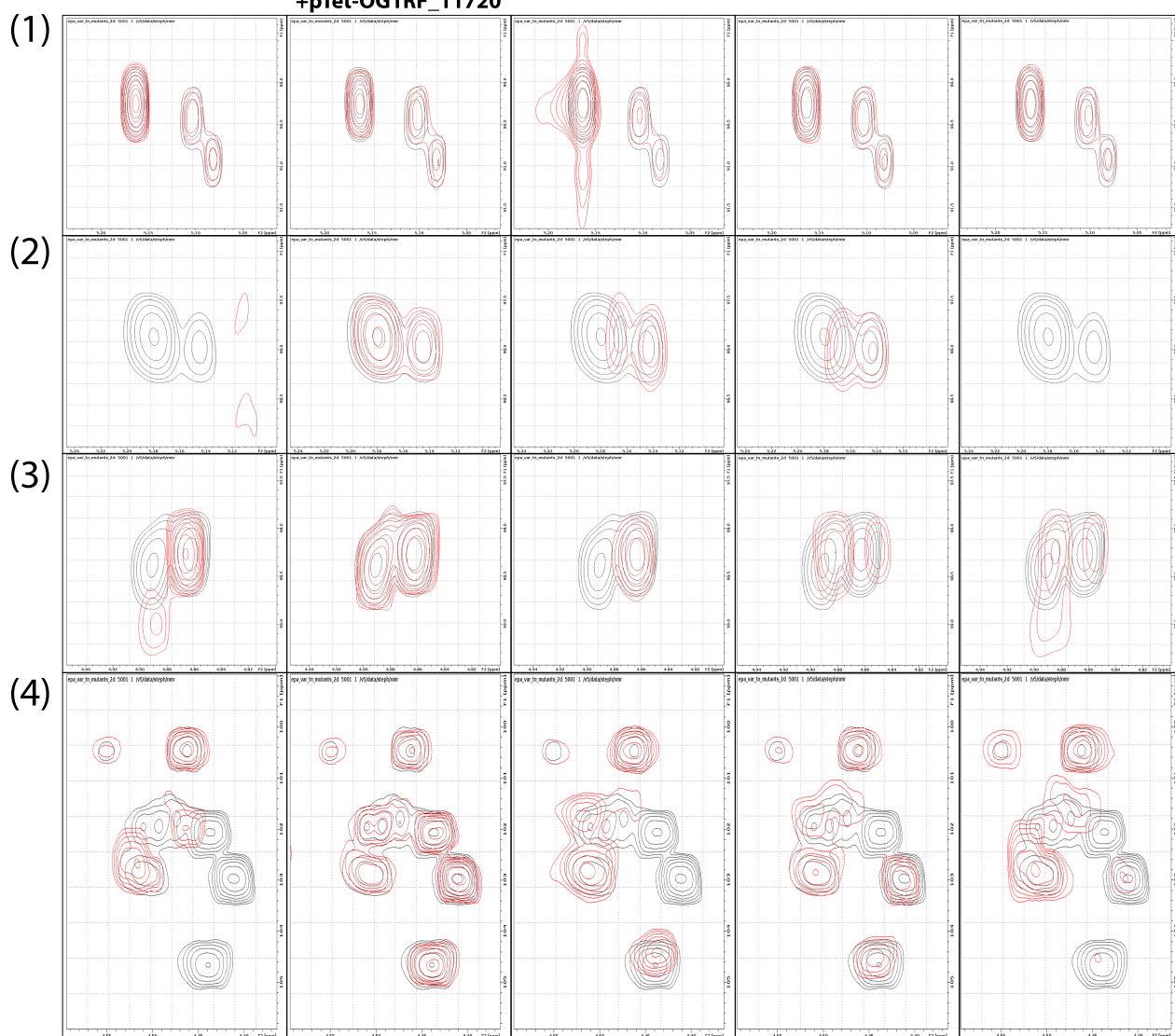

Supplement: S4 Fig — A. Region corresponding to anomeric protons (4.2–5.5 ppm) and anomeric carbons (90–105 ppm) highlighting four regions of the spectra (boxed) with signals shifted or changing in intensity in the epa mutants. B. Boxed regions in A. are shown for individual mutant and one complemented strain. (PDF) [file ppat.1007730.s004.pdf]

## Slide 1
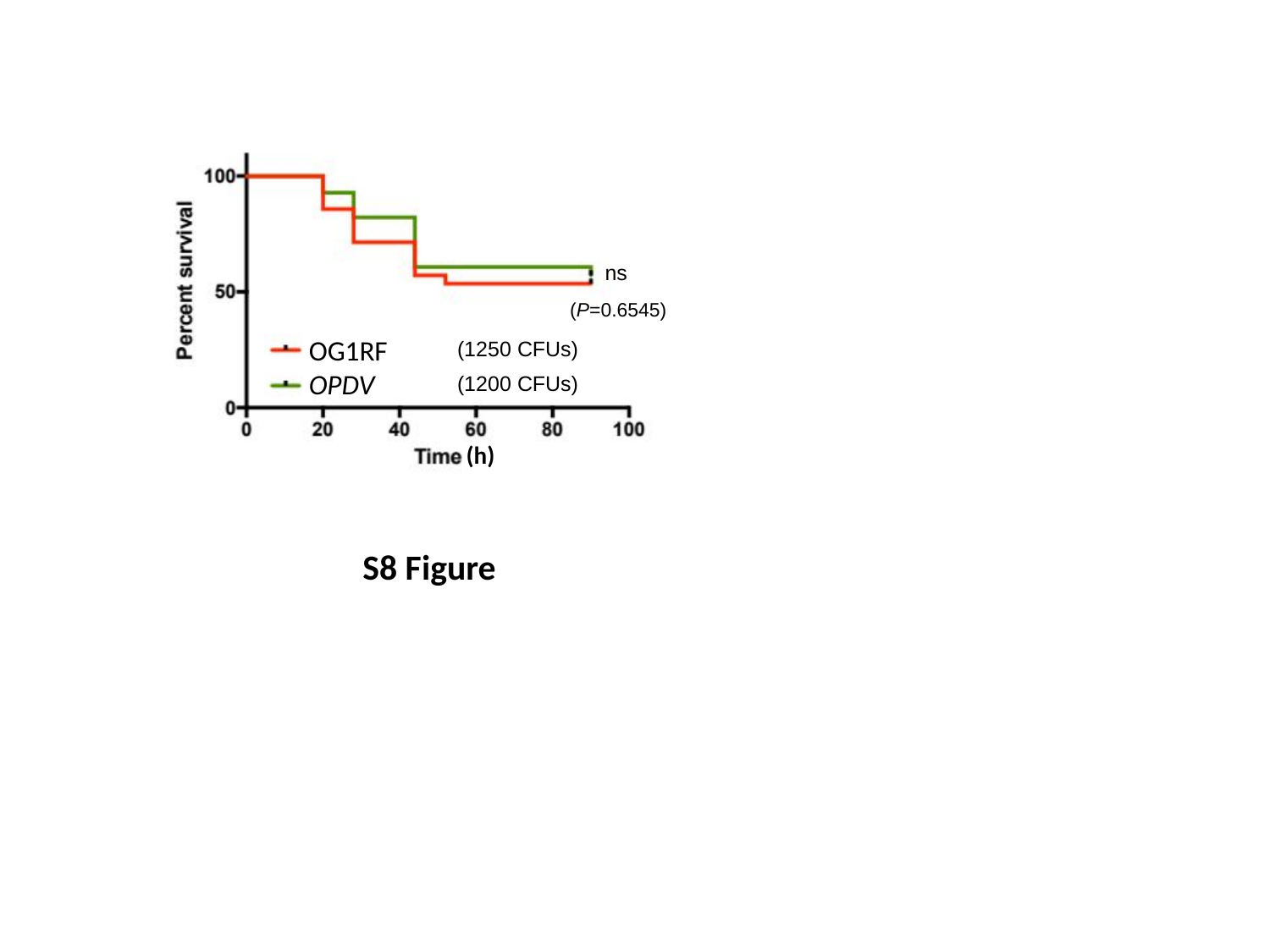

ns
(P=0.6545)
OG1RF
(1250 CFUs)
OPDV
(1200 CFUs)
(h)
S8 Figure

Supplement: S8 Fig — Survival of zebrafish larvae (n = 28) following infection with 1,000 CFUs of E. faecalis OG1RF (WT) and OPDV mutant was monitored over 90 h post infection. The lack of statistical significance (P = 0.645) was determined by Log-rank test. (PPTX) [file ppat.1007730.s008.pptx]
