# Supplementary material for: Decoration of the enterococcal polysaccharide antigen EPA is essential for virulence, cell surface charge and interaction with effectors of the innate immune system
Source: PLoS Pathog. 2019 May 2;15(5):e1007730. doi: 10.1371/journal.ppat.1007730 (PMC6497286; doi:10.1371/journal.ppat.1007730)
Supplement: S3 Fig — Following gel filtration, fractions containing neutral sugars were pooled and freeze-dried. EPA was hydrolysed in the presence of 4 N TFA at 100°C for 4 h. Monosaccharides were separated on a carbopac PA10 column by high performance anion exchange chromatography coupled to pulsed-amperometric detection. Representative chromatograms are shown for monosaccharide standards and each transposon mutant. EPA polysaccharides were extracted from three independent cultures to give average values in Fig 3C. (PPTX) [file ppat.1007730.s003.pptx]

## Slide 1
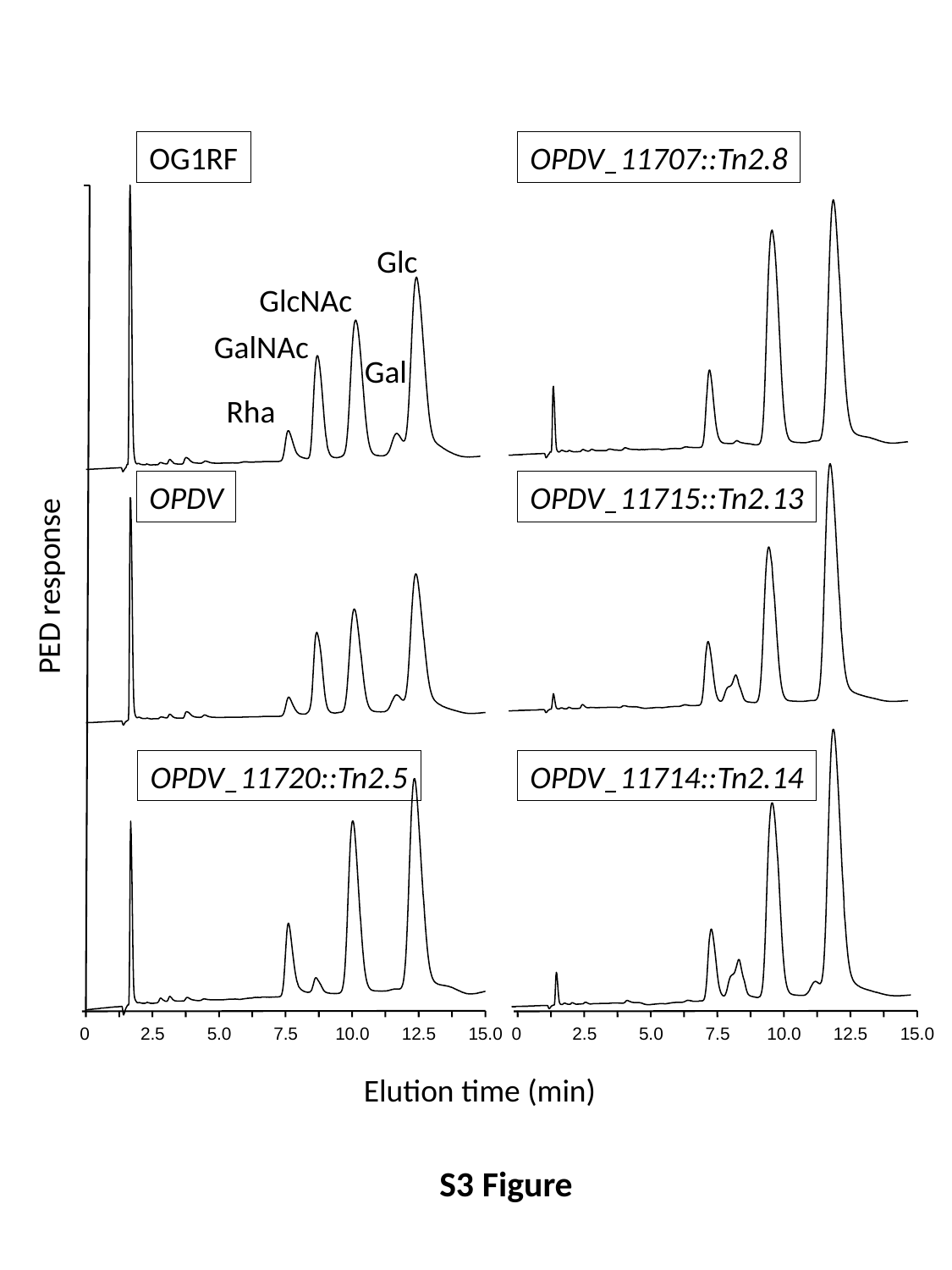

OG1RF
OPDV_11707::Tn2.8
Glc
GlcNAc
GalNAc
Gal
Rha
OPDV
OPDV_11715::Tn2.13
PED response
OPDV_11720::Tn2.5
OPDV_11714::Tn2.14
0
2.5
5.0
7.5
10.0
12.5
15.0
0
2.5
5.0
7.5
10.0
12.5
15.0
Elution time (min)
S3 Figure
