# Supplementary material for: Decoration of the enterococcal polysaccharide antigen EPA is essential for virulence, cell surface charge and interaction with effectors of the innate immune system
Source: PLoS Pathog. 2019 May 2;15(5):e1007730. doi: 10.1371/journal.ppat.1007730 (PMC6497286; doi:10.1371/journal.ppat.1007730)
Supplement: S5 Fig — (PPTX) [file ppat.1007730.s005.pptx]

## Slide 1
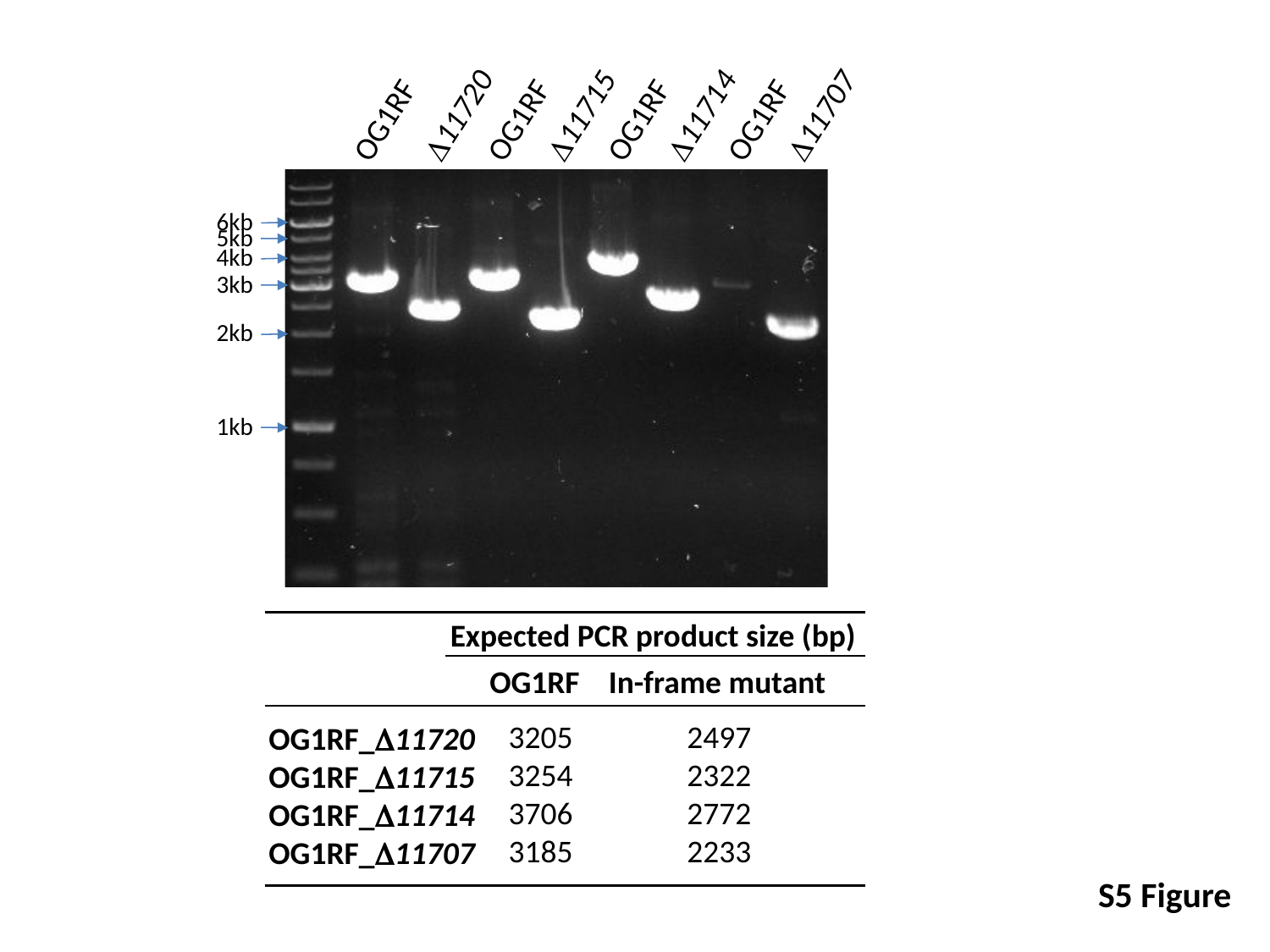

D11720
D11715
D11714
D11707
OG1RF
OG1RF
OG1RF
OG1RF
6kb
5kb
4kb
3kb
2kb
1kb
Expected PCR product size (bp)
OG1RF
In-frame mutant
3205
3254
3706
3185
2497
2322
2772
2233
OG1RF_D11720
OG1RF_D11715
OG1RF_D11714
OG1RF_D11707
S5 Figure
