# Supplementary material for: Decoration of the enterococcal polysaccharide antigen EPA is essential for virulence, cell surface charge and interaction with effectors of the innate immune system
Source: PLoS Pathog. 2019 May 2;15(5):e1007730. doi: 10.1371/journal.ppat.1007730 (PMC6497286; doi:10.1371/journal.ppat.1007730)
Supplement: S6 Fig — Peptidoglycan was extracted from three biological replicates, digested with mutanolysin, reduced, and disaccharide-peptides were separated by reverse-phase HPLC. The traces shown represent averaged values from the three independent replicates. The wild-type OG1RF muropeptide profile is shown for comparison, alongside the traces corresponding to each mutant and its complemented derivative: A, mutant OG1RF Δ11720; B, mutant OG1RF Δ11715; C, mutant OG1RF Δ11714; D, mutant OG1RF Δ11707. (PPTX) [file ppat.1007730.s006.pptx]

## Slide 1
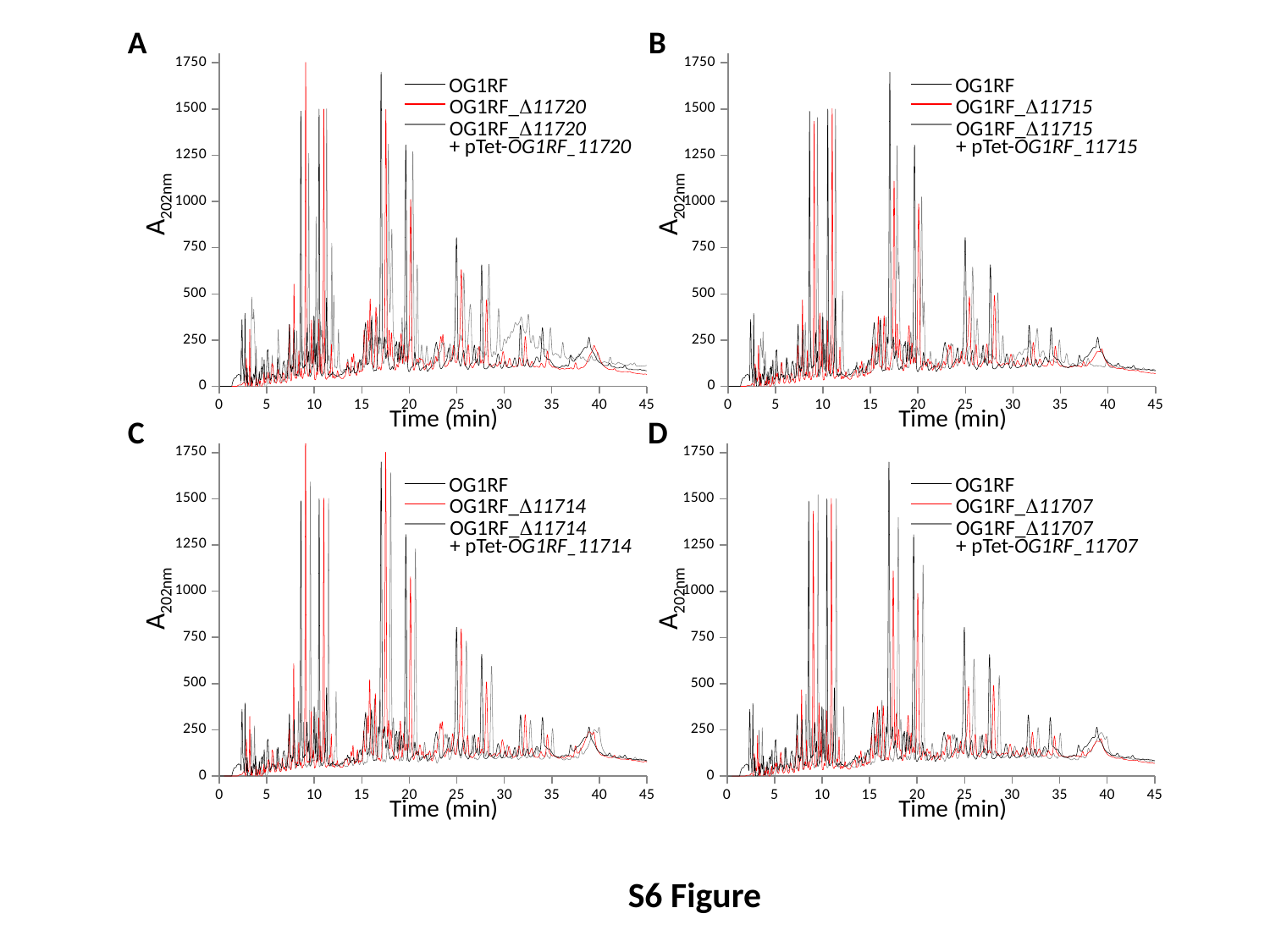

A
B
### Chart
| Category | OG1RF | | |
|---|---|---|---|
### Chart
| Category | OG1RF | | |
|---|---|---|---|OG1RF
OG1RF
OG1RF_D11720
OG1RF_D11715
OG1RF_D11720
+ pTet-OG1RF_11720
OG1RF_D11715
+ pTet-OG1RF_11715
A202nm
A202nm
Time (min)
Time (min)
C
D
### Chart
| Category | OG1RF | | |
|---|---|---|---|
### Chart
| Category | OG1RF | | |
|---|---|---|---|OG1RF
OG1RF
OG1RF_D11714
OG1RF_D11707
OG1RF_D11714
+ pTet-OG1RF_11714
OG1RF_D11707
+ pTet-OG1RF_11707
A202nm
A202nm
Time (min)
Time (min)
S6 Figure
