# Supplementary material for: Decoration of the enterococcal polysaccharide antigen EPA is essential for virulence, cell surface charge and interaction with effectors of the innate immune system
Source: PLoS Pathog. 2019 May 2;15(5):e1007730. doi: 10.1371/journal.ppat.1007730 (PMC6497286; doi:10.1371/journal.ppat.1007730)
Supplement: S7 Fig — Survival of zebrafish larvae (n>20) following infection with E. faecalis OG1RF (WT) and epa insertion mutant was monitored over 90 h post infection. A. Mutant OPDV_11720::Tn2.5. B. Mutant OPDV_11715::Tn2.13. C. Mutant OPDV_11714::Tn2.14. D. Mutant OPDV_11707::Tn2.8. Statistical significance was determined by Log-rank test; NS, P>0.05; **P<0.01; *** P<0.001; **** P<0.0001. The same data corresponding to the WT strain are shown in Fig 4A/4C and 4B/4D. (PPTX) [file ppat.1007730.s007.pptx]

## Slide 1
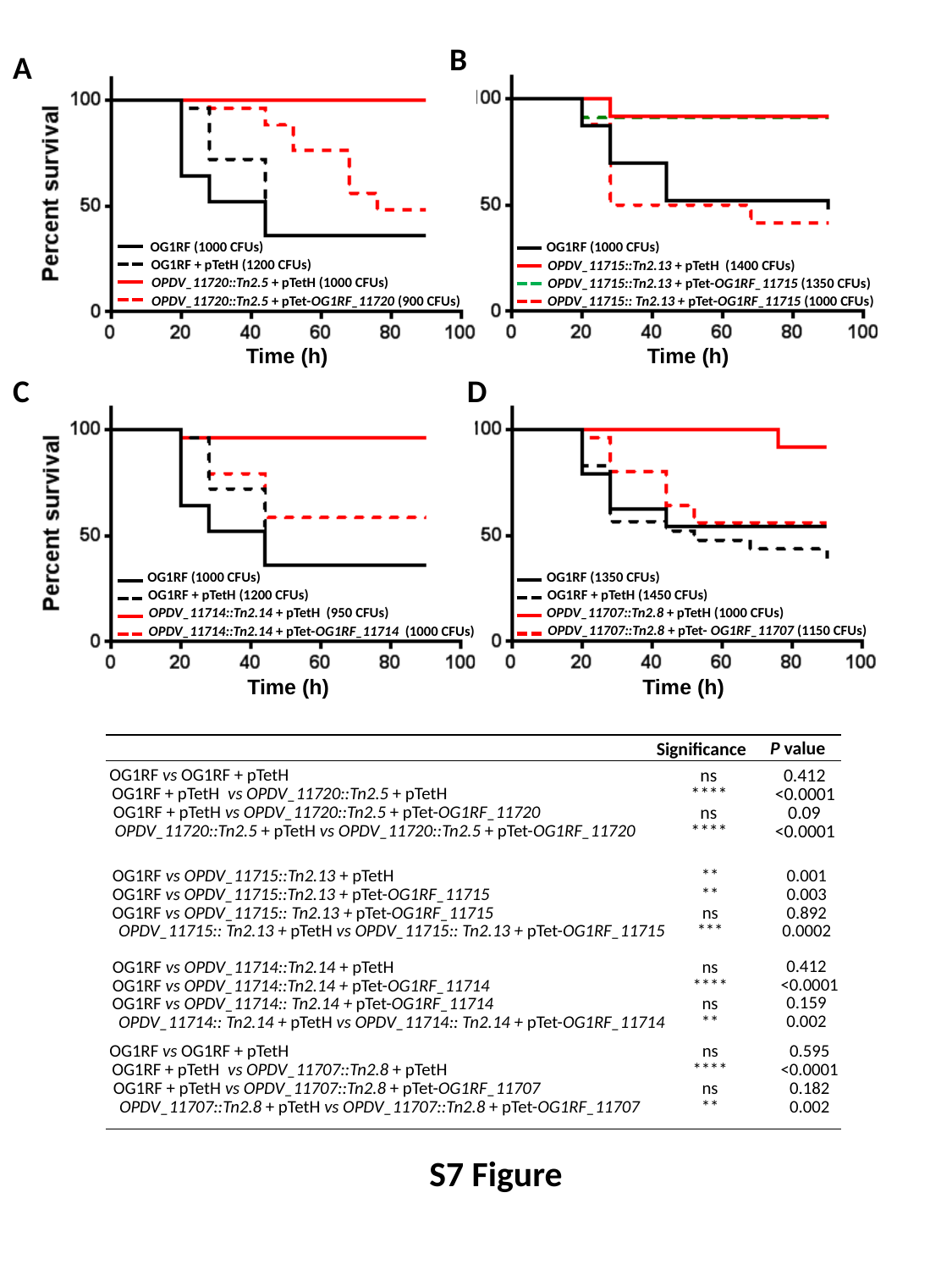

B
A
OG1RF (1000 CFUs)
OG1RF (1000 CFUs)
OPDV_11715::Tn2.13 + pTetH (1400 CFUs)
OG1RF + pTetH (1200 CFUs)
OPDV_11715::Tn2.13 + pTet-OG1RF_11715 (1350 CFUs)
OPDV_11720::Tn2.5 + pTetH (1000 CFUs)
OPDV_11715:: Tn2.13 + pTet-OG1RF_11715 (1000 CFUs)
OPDV_11720::Tn2.5 + pTet-OG1RF_11720 (900 CFUs)
Time (h)
Time (h)
C
D
OG1RF (1350 CFUs)
OG1RF (1000 CFUs)
OG1RF + pTetH (1450 CFUs)
OG1RF + pTetH (1200 CFUs)
OPDV_11707::Tn2.8 + pTetH (1000 CFUs)
OPDV_11714::Tn2.14 + pTetH (950 CFUs)
OPDV_11707::Tn2.8 + pTet- OG1RF_11707 (1150 CFUs)
OPDV_11714::Tn2.14 + pTet-OG1RF_11714 (1000 CFUs)
Time (h)
Time (h)
P value
0.412
<0.0001
0.09
<0.0001
Significance
ns
****
ns
****
OG1RF vs OG1RF + pTetH
OG1RF + pTetH vs OPDV_11720::Tn2.5 + pTetH
OG1RF + pTetH vs OPDV_11720::Tn2.5 + pTet-OG1RF_11720
OPDV_11720::Tn2.5 + pTetH vs OPDV_11720::Tn2.5 + pTet-OG1RF_11720
**
**
ns
***
0.001
0.003
0.892
0.0002
OG1RF vs OPDV_11715::Tn2.13 + pTetH
OG1RF vs OPDV_11715::Tn2.13 + pTet-OG1RF_11715
OG1RF vs OPDV_11715:: Tn2.13 + pTet-OG1RF_11715
OPDV_11715:: Tn2.13 + pTetH vs OPDV_11715:: Tn2.13 + pTet-OG1RF_11715
0.412
<0.0001
0.159
0.002
ns
****
ns
**
OG1RF vs OPDV_11714::Tn2.14 + pTetH
OG1RF vs OPDV_11714::Tn2.14 + pTet-OG1RF_11714
OG1RF vs OPDV_11714:: Tn2.14 + pTet-OG1RF_11714
OPDV_11714:: Tn2.14 + pTetH vs OPDV_11714:: Tn2.14 + pTet-OG1RF_11714
OG1RF vs OG1RF + pTetH
ns
****
ns
**
0.595
<0.0001
0.182
0.002
OG1RF + pTetH vs OPDV_11707::Tn2.8 + pTetH
OG1RF + pTetH vs OPDV_11707::Tn2.8 + pTet-OG1RF_11707
OPDV_11707::Tn2.8 + pTetH vs OPDV_11707::Tn2.8 + pTet-OG1RF_11707
S7 Figure
