# Supplementary material for: Decoration of the enterococcal polysaccharide antigen EPA is essential for virulence, cell surface charge and interaction with effectors of the innate immune system
Source: PLoS Pathog. 2019 May 2;15(5):e1007730. doi: 10.1371/journal.ppat.1007730 (PMC6497286; doi:10.1371/journal.ppat.1007730)
Supplement: S9 Fig — Cells from overnight cultures in BHI were diluted to an OD at 600 nm of 0.01 in 25 ml BHI and growth of standing cultures was monitored over 6 h. The data presented are the average of 3 independent cultures. The same OG1RF growth curves were used as a control in each graph. (PPTX) [file ppat.1007730.s009.pptx]

## Slide 1
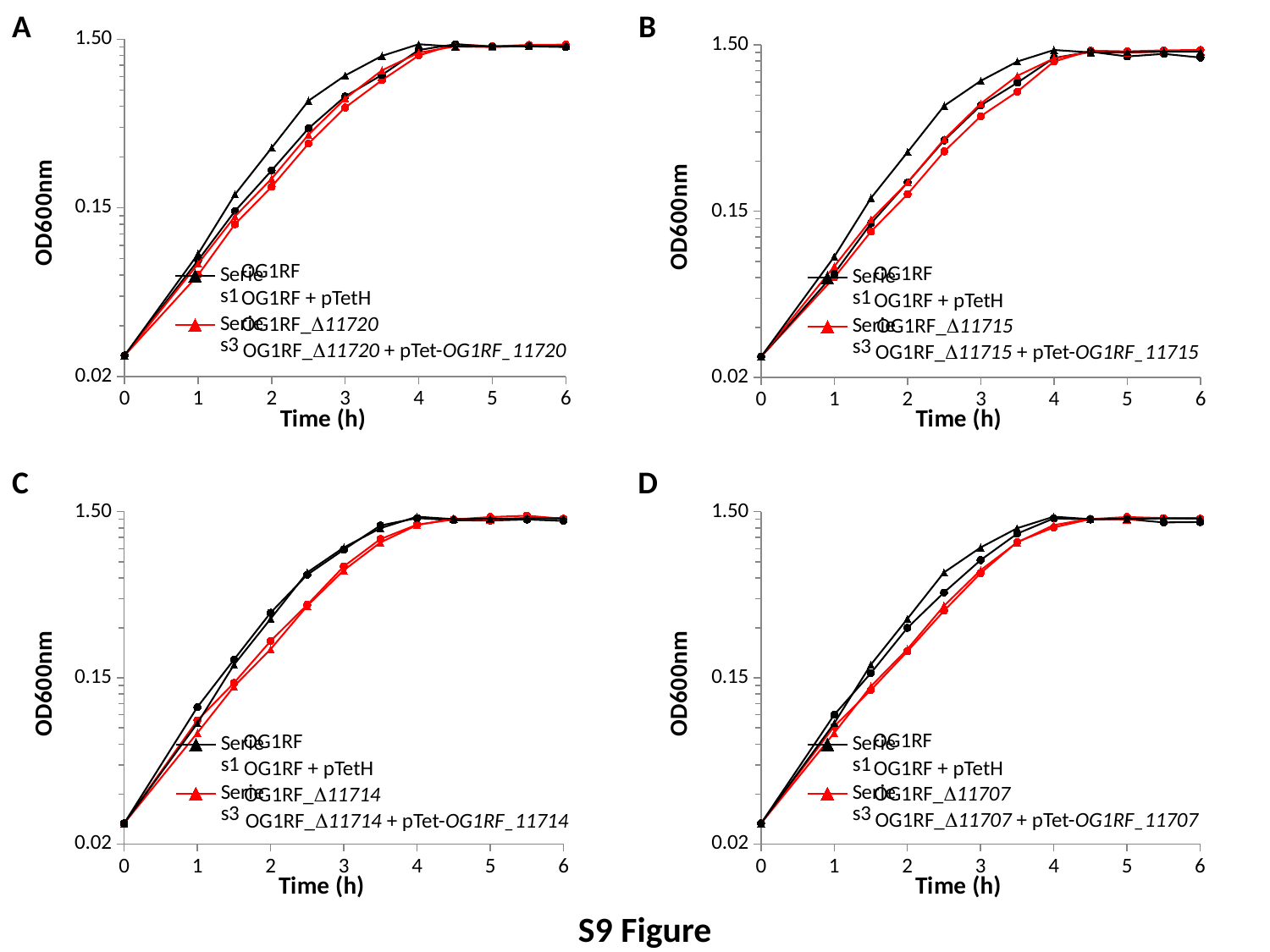

A
B
### Chart
| Category | | | | |
|---|---|---|---|---|
### Chart
| Category | | | | |
|---|---|---|---|---|OG1RF
OG1RF
OG1RF + pTetH
OG1RF + pTetH
OG1RF_D11720
OG1RF_D11715
OG1RF_D11720 + pTet-OG1RF_11720
OG1RF_D11715 + pTet-OG1RF_11715
C
D
### Chart
| Category | | | | |
|---|---|---|---|---|
### Chart
| Category | | | | |
|---|---|---|---|---|OG1RF
OG1RF
OG1RF + pTetH
OG1RF + pTetH
OG1RF_D11707
OG1RF_D11714
OG1RF_D11707 + pTet-OG1RF_11707
OG1RF_D11714 + pTet-OG1RF_11714
S9 Figure
