# Supplementary material for: Decoration of the enterococcal polysaccharide antigen EPA is essential for virulence, cell surface charge and interaction with effectors of the innate immune system
Source: PLoS Pathog. 2019 May 2;15(5):e1007730. doi: 10.1371/journal.ppat.1007730 (PMC6497286; doi:10.1371/journal.ppat.1007730)
Supplement: S10 Fig — A. Schematic representation of the mariner transposon used. It consists of a gentamycin resistance cassette flanked by two inverted repeats. B. Step 1: digestion of chromosomal DNA with SspI, which has a unique cleavage site in the gentamycin resistance cassette. C. step 2: self-ligation of SspI digestion products. D. step 3: reverse PCR on ligation products with two divergent oligonucleotides (Mar_dn and Mar_up). E. step 4: sequencing of the PCR product using oligonucleotide T7. (PPTX) [file ppat.1007730.s010.pptx]

## Slide 1
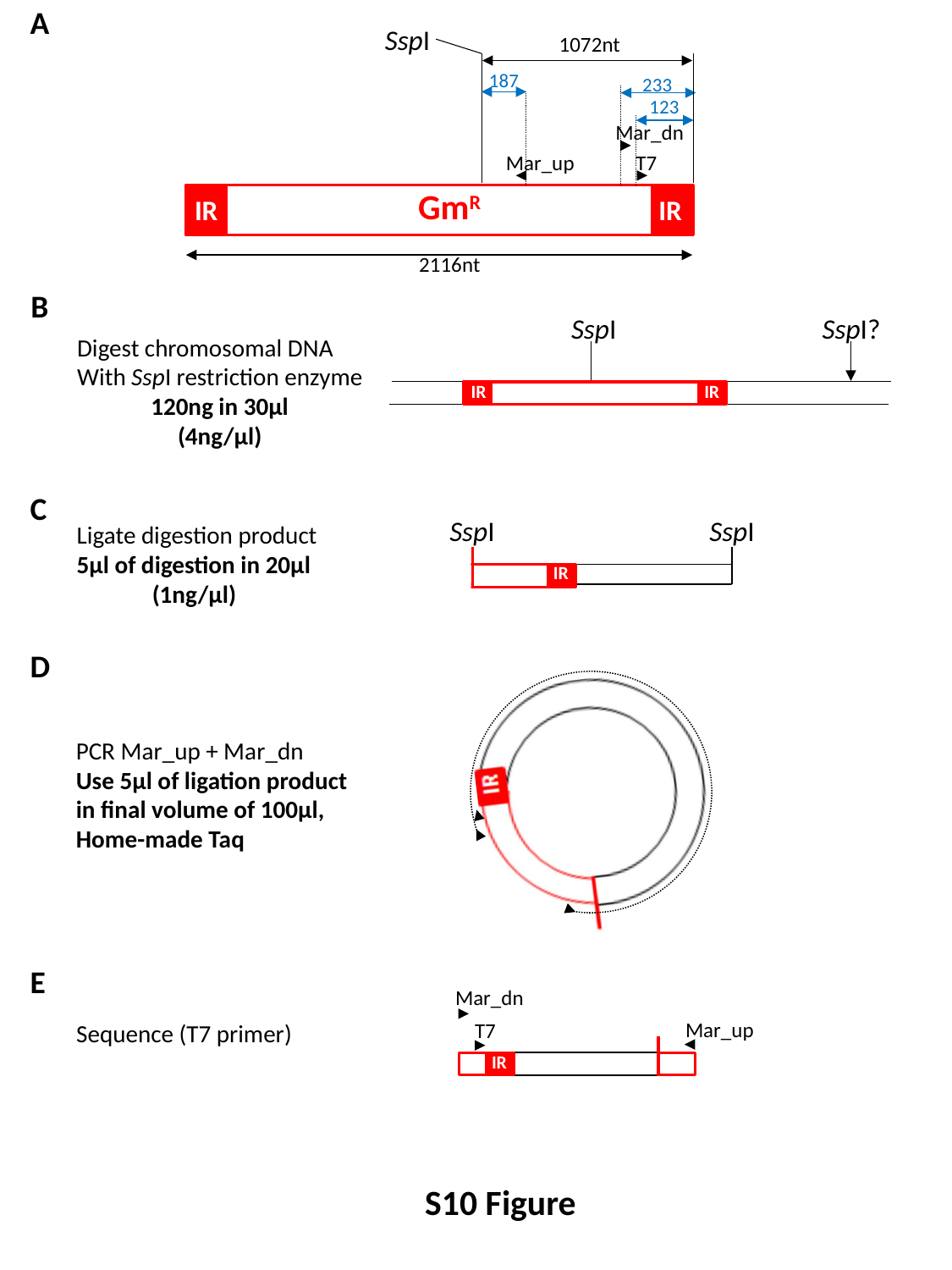

A
SspI
1072nt
233
187
123
Mar_dn
Mar_up
T7
GmR
IR
IR
2116nt
B
SspI
SspI?
Digest chromosomal DNA
With SspI restriction enzyme
120ng in 30µl
(4ng/µl)
IR
IR
C
SspI
SspI
Ligate digestion product
5µl of digestion in 20µl
(1ng/µl)
D
PCR Mar_up + Mar_dn
Use 5µl of ligation product
in final volume of 100µl,
Home-made Taq
E
Mar_dn
Sequence (T7 primer)
Mar_up
T7
IR
S10 Figure
