# Supplementary material for: Decoration of the enterococcal polysaccharide antigen EPA is essential for virulence, cell surface charge and interaction with effectors of the innate immune system
Source: PLoS Pathog. 2019 May 2;15(5):e1007730. doi: 10.1371/journal.ppat.1007730 (PMC6497286; doi:10.1371/journal.ppat.1007730)
Supplement: S1 Table — The values presented are the average of three independent biological replicates ± standard deviation. (DOCX) [file ppat.1007730.s011.docx]

**S1 Table. Electrophoretic mobility measurements (10^-8^ m²V^-1^s^-1^).** The values presented are the average of three independent biological replicates ± standard deviation. Each electrophoretic mobility value was measured from an average of 200 cells.

|  | | **pH** | | | | | | | | | | |
| --- | --- | --- | --- | --- | --- | --- | --- | --- | --- | --- | --- | --- |
| **Strains** | **2.0** | | | **3.0** | | **4.0** | | **4.5** | | **5.5** | |  |
| **OG1RF** | | | -2.11 ± 0.76 | | -3.29 ± 0.29 | | -3.48 ± 0.24 | | -3.22 ± 0.17 | | -2.65 ± 0.11 | |
| ***OPDV*** | | | -2.30 ± 0.12 | | -3.27 ± 0.06 | | -3.34 ± 0.13 | | -3.20 ± 0.13 | | -2.78 ± 0.10 | |
| ***OPDV_11720::Tn2.5*** | | | 0.29 ± 0.07 | | 0.01 ± 0.13 | | -0.80 ± 0.12 | | -1.00 ± 0.19 | | -1.49 ± 0.13 | |
| ***OPDV_11720::Tn2.5* + pTetH-*OG1RF_11720*** | | | -1.51 ± 0.52 | | -2.91 ± 0.27 | | -3.44 ± 0.26 | | -3.29 ± 0.18 | | -2.67 ± 0.06 | |
| ***OPDV_11707::Tn2.8*** | | | 0.18 ± 0.08 | | -0.38 ± 0.07 | | -1.27 ± 0.28 | | -1.51 ± 0.09 | | -1.88 ± 0.23 | |
| ***OPDV_11707::Tn2.8* + pTetH-*OG1RF_11707*** | | | -2.22 ± 0.17 | | -3.26 ± 0.11 | | -3.30 ± 0.18 | | -3.17 ± 0.13 | | -2.74 ± 0.08 | |
| ***OPDV_11715******::Tn2.13* (*epaOX*)** | | | 0.17 ± 0.05 | | -0.19 ± 0.08 | | -0.90 ± 0.01 | | -1.14 ± 0.10 | | -1.49 ± 0.07 | |
| ***OPDV_11715::Tn2.13* + pTetH-*OG1RF_11715*** | | | -2.28 ± 0.06 | | -3.15 ± 0.08 | | -3.27 ± 0.17 | | -3.17 ± 0.20 | | -2.68 ± 0.08 | |
| ***OPDV_11714::Tn2.14* (*epaX*)** | | | -1.05 ± 0.13 | | -3.02 ± 0.02 | | -3.55 ± 0.18 | | -3.26 ± 0.30 | | -2.81 ± 0.18 | |
| ***OPDV_11714::Tn2.14* + pTetH-*OG1RF_11714*** | | | -2.27 ± 0.13 | | -3.07 ± 0.16 | | -3.10 ± 0.18 | | -3.07 ± 0.11 | | -2.65 ± 0.13 | |
