# Supplementary material for: Decoration of the enterococcal polysaccharide antigen EPA is essential for virulence, cell surface charge and interaction with effectors of the innate immune system
Source: PLoS Pathog. 2019 May 2;15(5):e1007730. doi: 10.1371/journal.ppat.1007730 (PMC6497286; doi:10.1371/journal.ppat.1007730)
Supplement: S2 Table — The significance values have been calculated using two-way ANOVA. (DOCX) [file ppat.1007730.s012.docx]

**S2 Table. Statistical significance of pairwise comparisons of electrophoretic mobility with *OPDV*.** The significance values have been calculated using two-way ANOVA.

|  | **pH** | | | | |
| --- | --- | --- | --- | --- | --- |
| **Strains** | **2.0** | **3.0** | **4.0** | **4.5** | **5.5** |
| **OG1RF** | NS | NS | NS | NS | NS |
| ***OPDV_11720::Tn2.5*** | *** | *** | *** | *** | *** |
| ***OPDV_11720::Tn2.5* + pTetH-*OG1RF_11720*** | NS | NS | NS | NS | NS |
| ***OPDV_11707::Tn2.8*** | *** | *** | *** | *** | ** |
| ***OPDV_11707::Tn2.8* + pTetH-*OG1RF_11707*** | NS | NS | NS | NS | NS |
| ***OPDV_11715::Tn2.13* (*epaOX*)** | *** | *** | *** | *** | *** |
| ***OPDV_11715::Tn2.13* + pTetH-*OG1RF_11715*** | NS | NS | NS | NS | NS |
| ***OPDV_11714::Tn2.14* (*epaX*)** | *** | ** | NS | NS | NS |
| ***OPDV_11714::Tn2.14* + pTetH-*OG1RF_11714*** | NS | NS | NS | NS | NS |

NS, Not significant; ***, *P*<0.001; **, *P*<0.01
