# Supplementary material for: Decoration of the enterococcal polysaccharide antigen EPA is essential for virulence, cell surface charge and interaction with effectors of the innate immune system
Source: PLoS Pathog. 2019 May 2;15(5):e1007730. doi: 10.1371/journal.ppat.1007730 (PMC6497286; doi:10.1371/journal.ppat.1007730)
Supplement: S3 Table — (DOCX) [file ppat.1007730.s013.docx]

**S3 Table. Bacterial strains, plasmids and oligonucleotides used in this study.**

Strains, plasmids Relevant properties or genotype^a^ Source or reference

**Strains**

***Aerococcus viridans***

ATCC11563 [1]

***Bacillus megaterium***

KM [2]

***Bacillus subtilis***

168 [3]

***Enterococcus faecalis***

OG1RF Plasmid-free, virulent strain isolated from the oral cavity [4]

OG1RF OPDV OG1RF derivative with deletions in *oatA*, *pgdA*, *dltA* and *sigV* This work

***Enterococcus faecium***

DO (TX16) [5]

***Enterococcus hirae***

ATCC9790 [6]

***Escherichia coli***

TG1 Host for plasmid propagation NEB

TG1(*RepA*) TG1 derivative harboring *RepA* for pGhost propagation at 37°C P. Serror

***Listeria monocytogenes***

EGDe [7]

EGDe Δ*pgdA* [7]

***Streptococcus agalactiae***

NEM316 [8]

***Streptococcus gordonii***

DL-1 Challis Previously called *Streptococcus sanguis* [9]

***Streptococcus mutans***

UA159 [10]

***Streptococcus gallolyticus***

UCN34 [11]

**Plasmids**

pTetH pAT18 derivative for tetracycline-inducible expression in *E. faecalis* Lab stock

pGhost9 Thermosensitive plasmid used for gene replacement [12]

pGHH_11707 pGhost9 derivative used for in-frame deletion of *OG1RF_11707* This work

pGHH_11714 pGhost9 derivative used for in-frame deletion of *OG1RF_11714* This work

pGHH_11715 pGhost9 derivative used for in-frame deletion of *OG1RF_11715* This work

pGHH_11720 pGhost9 derivative used for in-frame deletion of *OG1RF_11720* This work

pMAD-Δ*oatA* pMAD derivative used to build the in-frame *oatA* deletion [13]

pMAD-Δ*pgdA* pMAD derivative used to build the in-frame *pgdA* deletion [13]

pMAD-Δ*dltA* pMAD derivative used to build the in-frame *dltA* deletion [14]

pMAD-Δ*sigV* pMAD derivative used to build the in-frame *sigV* deletion [14]

pTet-*OGR1F_11720* pTetH derivative encoding *OG1RF_11720* This work

pTet-*OGR1F_11715* pTetH derivative encoding *OG1RF_11715* This work

pTet-*OGR1F_11714* pTetH derivative encoding *OG1RF_11714* This work

pTet-*OGR1F_11707* pTetH derivative encoding *OG1RF_11707* This work

**Oligonucleotides^a^**

11720_Fw GGGCCATGGAAACAGCACTTGTTTCAATTATTATGC

11720_Rev TTTGGATCCTTCATTCTTTGCATATTTAAATGTTGTAT

11715_Fw CCCCCATGGAAAAAGAAAATTTAAAGTTAAGCGTGATTATTCC

11715_Rev TATGGATCCCTTCCCTAAAATTTTTCGATACATAAAATTATATA

11714_Fw AAACCATGGAAATTAGTATAATTGTTCCTGTTT

11714_Rev CCCGGATCCCTTATTAACCTTTCTTAAATACCTCTGTTGCA

11707_Fw AAACCATGGAAACATTCCTAATCACAGGCGGC

11707_Rev GGGGGATCCTTCTTTTAATTCATAATTAACATATTTATCCAAAC

11720_H11 CTTTGGCGAATTCTTGTTTATTATCTTTACTTAAAGGTC

11720_H12 CTATTCATTCTTTGCATACATAATAATTGAAACAAGTGCTGTTTCC

11720_H21 CAATTATTATGTATGCAAAGAATGAATAGGGAGGAAAAGGAAAAG

11720_H22 aaaCTCGAGGACATACATACTATAGTGATATGCTGAAACTTTTAAG

11715_H11 aaaCTCGAGAAGTCAAAGAGGCTATGTTCGGTCATC

11715_H12 CTTCCCTAACACATTATATACTGGAATAATCACGCTTAAC

11715_H21 ATTCCAGTATATAATGTGTTAGGGAAGTAAGGAGTTAAAGATGTCAG

11715_H22 aaaGGTACCCTTATTAAATCCACCTCTCAGTATAGCTAC

11714_H11 aaaGAATTCCATTTTGTACATGTTAAGTTCCTTGAACATG

11714_H12 CCTCTGTTGCATAACAGGAACAATTATACTAATTTCTGACATC

11714_H21 AATTGTTCCTGTTATGCAACAGAGGTATTTAAGAAAGGTTAATAAG

11714_H22 aaaCTCGAGCTAAGCAACTCTTTTTCTGTTAAAGCAAAC

11707_H11 aaaGAATTCGAGCTGATCATTCTATAGAAAGATGTACTTATG

11707_H12 GGACTGAATACTGCCGCCTGTGATTAGGAATGTTTCCAC

11707_H21 CTAATCACAGGCGGCAGTATTCAGTCCGGTTTGGATAAATATG

11707_H22 aaaGGTACCGTTTCTTAGTCTCTAAAAATACACGGCCAAC

^a^  restriction sites used for cloning are underlined (CCATGG, NcoI; GGATCC, BamHI; GAATTC, EcoRI;
 GGTACC, KpnI; CTCGAG, XhoI)

**References**

1. Nakel M, Ghuysen JM, Kandler O. Wall peptidoglycan in *Aerococcus viridans* strains 201 Evans and ATCC 11563 and in *Gaffkya homari* strain ATCC 10400. Biochemistry. 1971;10(11):2170-2175. pmid: 4254570.

2. Foster SJ, Johnstone K. The use of inhibitors to identify early events during *Bacillus megaterium* KM spore germination. Biochem J. 1986;237(3):865-870. pmid: 3099759.

3. Kunst F, Ogasawara N, Moszer I, Albertini AM, Alloni G, Azevedo V, et al. The complete genome sequence of the gram-positive bacterium *Bacillus subtilis*. Nature. 1997;390(6657):249-256. doi: 10.1038/36786. pmid: 9384377.

4. Dunny GM, Brown BL, Clewell DB. Induced cell aggregation and mating in *Streptococcus faecalis*: evidence for a bacterial sex pheromone. Proc Natl Acad Sci U S A. 1978;75(7):3479-3483. pmid: 98769.

5. Qin X, Galloway-Pena JR, Sillanpaa J, Roh JH, Nallapareddy SR, Chowdhury S, et al. Complete genome sequence of *Enterococcus faecium* strain TX16 and comparative genomic analysis of *Enterococcus faecium* genomes. BMC Microbiol. 2012;12:135. doi: 10.1186/1471-2180-12-135. pmid: 22769602.

6. Gaechter T, Wunderlin C, Schmidheini T, Solioz M. Genome sequence of *Enterococcus hirae* (*Streptococcus faecalis*) ATCC 9790, a model organism for the study of ion transport, bioenergetics, and copper homeostasis. J Bacteriol. 2012;194(18):5126-5127. doi: 10.1128/JB.01075-12. pmid: 22933757.

7. Boneca IG, Dussurget O, Cabanes D, Nahori MA, Sousa S, Lecuit M, et al. A critical role for peptidoglycan *N*-deacetylation in *Listeria* evasion from the host innate immune system. Proc Natl Acad Sci U S A. 2007;104(3):997-1002. doi: 0609672104 [pii]

10.1073/pnas.0609672104. pmid: 17215377.

8. Glaser P, Rusniok C, Buchrieser C, Chevalier F, Frangeul L, Msadek T, et al. Genome sequence of *Streptococcus agalactiae*, a pathogen causing invasive neonatal disease. Mol Microbiol. 2002;45(6):1499-1513. pmid: 12354221.

9. Kilian M, Mikkelsen L, Henrichsen J. Taxonomic study of viridans streptococci: description of *Streptococcus gordonii* sp. nov. and emended descriptions of *Streptococcus sanguis* (White and Niven 1946), *Streptococcus oralis* (Bridge and Sneath 1982), and *Streptococcus mitis* (Andrewes and Horder 1906). International Journal of Systematic and Evolutionary Microbiology. 1989;39(4):471-484.

10. Ajdic D, McShan WM, McLaughlin RE, Savic G, Chang J, Carson MB, et al. Genome sequence of *Streptococcus mutans* UA159, a cariogenic dental pathogen. Proc Natl Acad Sci U S A. 2002;99(22):14434-14439. doi: 10.1073/pnas.172501299. pmid: 12397186.

11. Rusniok C, Couve E, Da Cunha V, El Gana R, Zidane N, Bouchier C, et al. Genome sequence of *Streptococcus gallolyticus*: insights into its adaptation to the bovine rumen and its ability to cause endocarditis. J Bacteriol. 2010;192(8):2266-2276. doi: 10.1128/JB.01659-09. pmid: 20139183.

12. Maguin E, Duwat P, Hege T, Ehrlich D, Gruss A. New thermosensitive plasmid for gram-positive bacteria. J Bacteriol. 1992;174(17):5633-5638. pmid: 1324906.

13. Hebert L, Courtin P, Torelli R, Sanguinetti M, Chapot-Chartier MP, Auffray Y, et al. *Enterococcus faecalis* constitutes an unusual bacterial model in lysozyme resistance. Infect Immun. 2007;75(11):5390-5398. doi: IAI.00571-07 [pii] 10.1128/IAI.00571-07. pmid: 17785473.

14. Le Jeune A, Torelli R, Sanguinetti M, Giard JC, Hartke A, Auffray Y, et al. The extracytoplasmic function sigma factor SigV plays a key role in the original model of lysozyme resistance and virulence of *Enterococcus faecalis*. PLoS ONE. 2010;5(3):e9658. doi: 10.1371/journal.pone.0009658. pmid: 20300180.
